# Combinatorial conditioning of adipose derived‐mesenchymal stem cells enhances their neurovascular potential: Implications for intervertebral disc degeneration

**DOI:** 10.1002/jsp2.1072

**Published:** 2019-12-19

**Authors:** Abbie. L. A. Binch, Stephen M. Richardson, Judith A. Hoyland, Frank P. Barry

**Affiliations:** ^1^ Regenerative Medicine Institute (REMEDI), National University of Ireland Galway (NUI Galway) Galway Ireland; ^2^ Division of Cell Matrix Biology and Regenerative Medicine, School of Biological Sciences, Faculty of Biology, Medicine and Health Manchester Academic Health Sciences Centre, University of Manchester Manchester UK; ^3^ NIHR Manchester Biomedical Research Centre, Central Manchester Foundation Trust, Manchester Academic Health Science Centre Manchester UK

**Keywords:** disc degeneration, intervertebral disc, mesenchymal stem cells, regenerative therapy, stem cells

## Abstract

**Background:**

Mesenchymal stem cells (MSCs) are becoming an increasingly attractive option for regenerative therapies due to their availability, self‐renewal capacity, multilineage potential, and anti‐inflammatory properties. Clinical trials are underway to test the efficacy of stem cell‐based therapies for the repair and regeneration of the degenerate intervertebral disc (IVD), a major cause of back pain. Recently, both bone marrow‐derived MSCs and adipose‐derived stem cells (ASCs) have been assessed for IVD therapy but there is a lack of knowledge surrounding the optimal cell source and the response of transplanted cells to the low oxygen, pro‐inflammatory niche of the degenerate disc. Here, we investigated several neurovascular factors from donor‐matched MSCs and ASCs that may potentiate the survival and persistence of sensory nerve fibers and blood vessels present within painful degenerate discs and their regulation by oxygen tensions and inflammatory cytokines.

**Methods:**

Donor‐matched ASCs and MSCs were conditioned with either IL‐1β or TNFα under normoxic (21% O_2_) or hypoxic (5% O_2_) conditions. Expression and secretion of several potent neurovascular factors were assessed using qRT‐PCR and human magnetic Luminex assay.

**Results:**

ASCs and MSCs expressed constitutive levels of key neurotrophic factors; and stimulation of ASCs with hypoxia triggered increased secretion of both angiogenic factors (Ang‐2 and VEGF‐A) and neurotrophic (NGF and NT‐3) compared to MSCs. We also report increased transcriptional regulation of pain‐associated neuropeptides in hypoxia stimulated ASCs compared to those in normoxic conditions. We demonstrate transcriptional and translational upregulation of NGF, NT‐3, Ang‐1, and FGF‐2 in response to cytokines in ASCs in 21% and 5% O_2_.

**Conclusions:**

This work highlights fundamental differences between the neurovascular secretome of donor‐matched ASCs and MSCs, demonstrating the importance of cell‐selection for tissue specific regeneration to reduce ectopic sensory nerve and blood vessel survival and improve patient outcomes.

## Graphical abstract

The objectives of this study were to establish whether there is a difference in expression of angiogenic and neurotrophic factors between undifferentiated donor‐matched ASCs and MSCs and whether the expression of these factors is affected by culture in a hypoxic or inflammatory environment alone, or in combination. The data presented here highlights increased responsiveness of ASCs to cytokine stimulation under low oxygen by enhancing secretion of factors involved in neurogenesis (NGF and NT‐3) and neoangiogenesis (VEGF‐A and Ang‐1)within the IVD; a process which could lead to persistent discogenic pain.

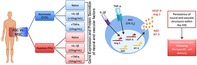

**Abbreviations**
AF: annulus fibrosus
Ang‐1: angiopoietin‐1
Ang‐2: angiopoietin‐2
ASCs: adipose‐derived mesenchymal stem cells
BDNF: brain‐derived neurotrophic factor
CGRP: calcitonin gene related peptide
ELISA: enzyme‐linked immunosorbent assay
FGF‐2: fibroblastic growth factor
IVD: intervertebral disc
IDD: intervertebral disc degeneration
IL‐1β: interleukin‐1 beta
LBP: low back pain
MSCs: mesenchymal stem cells
NP: nucleus pulposus
NGF: nerve growth factor
NT‐3: neurotrophin‐3
qRT‐PCR: quantitative real‐time polymerase chain reaction
TNFα: tumor necrosis factor alpha
VEGF‐A: vascular endothelial growth factor‐A

## 1 BACKGROUND

In recent years, mesenchymal stem cell (MSC)‐based therapies have been tested in a wide array of degenerative conditions, including musculoskeletal diseases, Crohn's disease, complications of diabetes, and others. There is a growing clinical database and available results support the preliminary conclusion that these strategies can provide an effective regenerative or disease‐modifying outcome. A definitive conclusion will become available when late stage clinical trials are completed. Pain is a key feature of many musculoskeletal conditions, including, for example, chronic severe lower back pain associated with intervertebral disc (IVD) disease (IDD) or chronic joint pain in osteoarthritis. The impact on patients is immense and leads to reduced quality of life and sometimes serious addictions to opioid medications.

Low back pain (LBP) is an increasingly common health problem and a major cause of disability worldwide, resulting in its inclusion in the World Health Organization's list of 12 priority diseases. Total annual costs associated with back pain are estimated in excess of €12 billion across Europe. Both the UK and the US report LBP as being the most common cause of disability amongst young adults, leading to a loss of more than 100 and 149 million workdays per year, respectively.^1^ It is starkly clear that as the world population ages, the disease burden of LBP will increase substantially. The causes of LBP remain multifactorial, although approximately 40% of all cases are attributed to the degeneration of one or more IVDs.^2^

Current treatments include conservative symptomatic pain relief or end‐stage surgical treatments that are often ineffective as they are symptom‐, rather than disease‐modifying.^3^, ^4^, ^5^ Thus, regenerative therapies targeting the degenerate IVD have emerged at the forefront of research within the spinal field; with considerable attention focusing on the development of cell‐based therapeutics that address the underlying pathogenesis of degenerative disc disease.^6^, ^7^, ^8^ Many groups have studied the potential use of MSCs from bone‐marrow or adipose (ASCs) to repair and regenerate the damaged IVD, either alone or in combination with native NP cells, sometimes delivered using biomaterial scaffolds and carriers.^9^, ^10^ Stem cells are an attractive option for regenerative therapies due to their availability, self‐renewal capacity, multilineage potential, and immunosuppressive properties. One particular question is the source of stem cells, and various studies have investigated the differences between MSCs and ASCs for their regenerative potential.

Clinical trials are underway to elucidate a successful stem cell‐based therapy for the repair and regeneration of IVD tissue, with preclinical and phase I data showing promising results for the safety and efficacy of allogeneic and autologous ASCs and MSCs.^11^, ^12^, ^13^, ^14^, ^15^ Although cell‐therapies have been reported to increase hydration and flexibility of the affected disc, reduce pain, and in some instances, increase disc height, there is a subset of patients who do not show improvement in pain score, thus failing to meet primary endpoints. The causes of patient nonresponse are inconclusive, yet it could be suggested that the delivered MSCs respond negatively to the degenerate microenvironment resulting in persistent pain following delivery.^11^, ^13^ While several in vivo studies have demonstrated MSC engraftment and long‐term survival in the harsh environment of normal disc tissue,^16^, ^17^, ^18^, ^19^, ^20^ the capacity of MSCs and ASCs to stimulate the survival and persistence of nerve and blood vessels within painful discs has not been addressed.

This study aimed to test the hypothesis that hypoxic and pro‐inflammatory conditioning mimicking that of the degenerate IVD, would modify the expression and secretory profile of donor‐matched ASCs and MSCs by enhancing the expression of potent angiogenic and neurotrophic factors, thus exacerbating pain in patients with innervated discs.

Our objectives were to establish whether there is a difference in expression of angiogenic and neurotrophic factors between undifferentiated ASCs and MSCs and whether the expression of these factors is affected by culture in a hypoxic or inflammatory environment alone, or in combination.

To the authors' knowledge, this is the first study to investigate and report fundamental differences in the neurovascular expression and secretory profile of ASCs and MSCs, identifying an optimal cell source for regenerative therapies of the IVD to reduce the occurrence of neural and vascular growth and improve clinical outcomes.

## 2 METHODS

### 2.1 Isolation and culture of MSCs and ASCs

All procedures and experiments were performed with relevant National Research Ethics Service and University of Manchester approvals. Bone marrow from proximal femur and subcutaneous adipose tissue samples were obtained from three donors (one male, two females, mean age 50 years, range 40‐62 years) undergoing hip replacement surgery who provided written informed consent.

Cells were isolated from bone marrow and adipose tissue as previously described.^21^ Briefly, bone marrow aspirates were washed with PBS and then centrifuged to obtain a cell pellet, and the pellet was resuspended in Minimum Essential Medium α‐modification (αMEM; Sigma‐Aldrich) which included nonessential amino acids, 110 mg/L sodium pyruvate, 1000 mg/L glucose supplemented with final concentrations of 100 U/mL penicillin, 100 μg/mL streptomycin and 0.25 μg/mL amphotericin, 2 mM GlutaMAX (Life Technologies), and 20% (vol/vol) fetal bovine serum (FBS). MSCs were isolated using density gradient centrifugation as previously described.^21^, ^22^ Adipose tissue was finely minced with a scalpel and then ASCs were isolated enzymatically as previously described.^23^ Both cell types were maintained at 37°C in a humidified atmosphere containing 5% (vol/vol) CO_2_; the culture medium was changed after 5 days and cells cultured to ∼80% confluence in complete αMEM as above supplemented with 10% FBS. MSC and ASC phenotype was confirmed as previously described (data not shown).^21^, ^23^, ^24^

### 2.2 Cytokine regulation of angiogenic factors, neurotrophic factors, and pain peptides in donor‐matched adipose and bone‐marrow‐derived MSCs

Following expansion to passage three, ASCs and MSCs were plated into wells of a 12‐well culture plate at a density of 50 000 cells per well. The following day cells were washed and exposed to low serum medium (complete medium as above supplemented with 2% FCS) either alone or with the addition of IL‐1β (10 ng/mL) or TNFα (10 ng/mL) for 48 hours in either 21% or 5% O_2_. All conditions were performed in technical triplicate. Following 48 hours conditioned medium was collected and stored at −80°C until required for further analysis.

### 2.3 Metabolic activity of MSCs under different oxygen conditions and cytokine stimulations

The metabolic cellular activity of ASCs and MSCs cultured at either 21% or 5% O_2_ was assessed using Alamar Blue assay (Thermo) in low serum media (2% FBS) at day 0 and day 2 as per the manufacturers protocol. The absorbance was recorded using a fluorescence microplate reader (TECAN) fluorescence excitation and emission wavelengths of 590/540 nm. Fluorescence was recorded for cellular constructs and normalized to the fluorescence of day 0 samples as an indication of cytotoxicity/proliferation.

### 2.4 Quantitative real‐time PCR

After 48 hours in normoxic or hypoxic culture with or without cytokine stimulation RNA was extracted using PureLink RNeasy Spin Columns (Invitrogen) as per manufacturer's instructions and quantification performed using the NanoDrop (Thermo). cDNA was synthesized using the High‐Capacity cDNA Reverse Transcription Kit (Applied Biosystems) as per manufacturer's instructions and diluted to a final concentration of 5 ng/μL for use in quantitative real‐time polymerase chain reaction (qRT‐PCR).

qRT‐PCR was performed on a StepOnePlus Real‐Time PCR System (Applied Biosystems) in order to investigate gene expression levels of angiogenic factors (Angiopoietin‐1 [Ang‐1], Ang‐2, basic fibroblastic growth factor [FGF‐2], and vascular endothelial growth factor‐A [VEGF‐A]); neurotrophic factors (nerve growth factor [NGF], brain‐derived neurotrophic factor [BDNF], and neurotrophin‐3 [NT‐3]), and pain‐related peptides (substance P and calcitonin gene related peptide [CGRP]).

Reactions were prepared in duplicate using Fast SYBR Green Master Mix (Applied Biosystems, UK) and predesigned KiCqStart Primers (Sigma‐Aldrich; Table 1) to a total volume of 10 μL, containing 10 ng cDNA and 600 nM each primer. Data were analyzed according to the 2^−ΔCt^ method, with expression normalized to 18S reference gene.

**Table 1:** Primer sequences used in qRT‐PCR reactions

Gene	Forward primer (5′➔3′)	Reverse Primer (5′➔3′)	Accession number
*18S*	ATCGGGGATTGCAATTATTC	CTCACTAAACCATCCAATCG	NM_003286
*NGF*	GGTGCATAGCGTAATGTC	TGAAGTTTAGTCCAGTGGG	NM_002506
*BDNF*	CAAAAGTGGAGAACATTTGC	AACTCCAGTCAATAGGTCAG	NM_001143811
*NT‐3*	CGATCTTACAGGTGAACAAG	AATTTTCCTTAACGTCCACC	NM_001102654
*Ang‐1*	ATGTTAACAGGAGGATGGTG	GAAGTAGTGCCACTTTATCC	NM_001199859
*Ang‐2*	AAGAGAAAGATCAGCTACAGG	CCTTAGAGTTTGATGTGGAC	NM_001118887
*FGF‐2*	TGGCTTCTAAATGTGTTACG	GTTTATACTGCCCAGTTCG	NM_002006
*VEGF‐A*	AATGTGAATGCAGACCAAAG	GACTTATACCGGGATTTCTTG	NM_001204384
*Substance P*	ATTCTGTGGCTTATGAAAGG	CATTGACACAAATGAAGCTG	NM_003182
*CGRP*	CAGCTGAATGACTCAAGAAG	GATGCACAATAGGTAACTGC	NM_001033953

### 2.5 Magnetic Luminex Assay analysis of conditioned medium of cytokine stimulated donor‐matched MSCs under normoxic and hypoxic conditions

Levels of Ang‐1, Ang‐2, FGF‐2, and VEGF‐A, NGF, BDNF, and NT‐3, were measured in cell culture supernatant from three patients in technical duplicates using quantitative Luminex assays (R&D Systems) according the manufacturer's instructions, and analyzed using the Biorad Bioplex 200 multiplex ELISA system.

### 2.6 Enzyme‐linked immunosorbent assay

Substance P levels were measured in cell culture supernatant from three patients in technical duplicate using an ELISA for detection of human substance P (Abcam) as per manufacturer's instructions and the absorbance read at 450 nm and 570 nm using a Victor X3 plate reader (PerkinElmer).

### 2.7 Statistical analysis

Data were assessed for normality using the Shapiro‐Wilks test and found to be nonparametric. Statistical significance determined using two‐way ANOVA with Tukey's multiple comparisons test, * = *P* ≤ .05.

## 3 ETHICAL APPROVAL AND CONSENT TO PARTICIPATE

All procedures and experiments were performed with relevant National Research Ethics Service and University of Manchester approvals. Bone marrow and subcutaneous adipose tissue samples were obtained from three donors undergoing hip replacement surgery who provided written informed consent.

## 4 RESULTS

### 4.1 Metabolic activity of ASCs and MSCs was not affected by hypoxic conditions or cytokine stimulation

**Figure 1:**
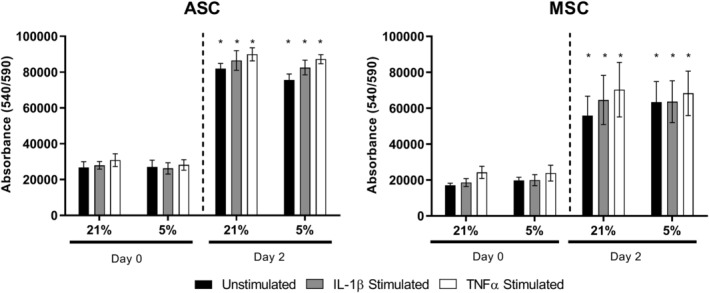
Metabolic activity of ASCs and MSCs exposed to cytokines IL‐1β or TNFα under 21% or 5% oxygen. AlamarBlue assessment of metabolic activity of human donor‐matched ASCs (A) and MSCs (B) under either 21% or 5% O_2_ and stimulated with either 10 ng/ml of IL‐1β or TNFα for 48 hours. n = 3. Values represent the mean ± SEM. Statistical significance determined using two‐way ANOVA with Tukey's multiple comparisons test, * = *P* ≤ .05 relative to corresponding group on day 0

As a method of confirming cell viability and proliferation, we examined the metabolic activity of MSCs under different treatment conditions. Metabolic activity of ASCs (Figure 1A) and MSCs (Figure 1B) was significantly increased after 48 hours under 21% and 5% O_2_ irrespective of cytokine stimulation compared to day 0. There were no significant differences between unstimulated controls and cytokine stimulated groups at the same time points and there were no significant differences between cells cultured under 21% or 5% O_2_ conditions at the same time points. Thus, no changes in metabolic activity of cells were observed due to oxygen levels or cytokine concentration after 48 hours.

### 4.2 Angiogenic factor expression in ASCs and MSCs in response to low oxygen and inflammatory cytokines

**Figure 2:**
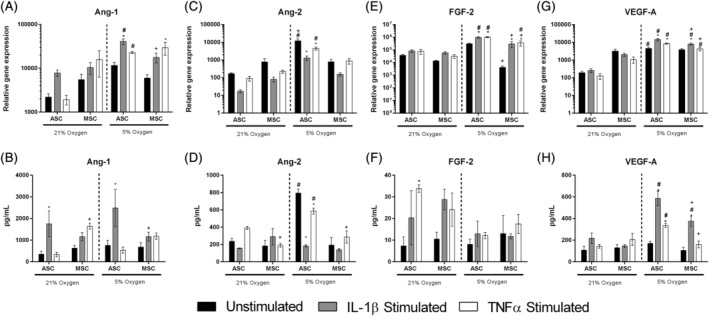
Gene and protein regulation of pro‐angiogenic factors in human donor‐matched ASCs and MSCs exposed to cytokines IL‐1β or TNFα under 21% or 5% oxygen. Pro‐angiogenic factors Ang‐1 (A,B), Ang‐2 (C, D), FGF‐2 (E, F) and VEGF‐A (G, H) mRNA expression (A, C, E, G) and levels of secreted protein (B, D, F, H) in human ASCs and MSCs stimulated with either IL‐1β or TNFα under different oxygen concentrations for 48 hours. Gene expression was normalized to that of reference gene 18S. Values represent the mean ± SEM. Statistical significance determined using two‐way ANOVA with Tukey's multiple comparisons test, * = *P* ≤ .05 (* = significant compared to unstimulated control within the same group; # = significant compared to corresponding sample in 21% oxygen; + = significance between MSCs and ASCs of the same treatment; n = 3)

The expression and secretion of several angiogenic factors from donor‐matched MSCs were assessed following stimulation with cytokines under different oxygen concentrations. We report that Ang‐1 expression from ASCs was significantly upregulated at gene and protein level by IL‐1β under 5% O_2_ (Figure 2A,B) and at protein level only under 21% O_2_ (Figure 2B). Ang‐1 was significantly upregulated by TNFα in MSCs under 5% O_2_ compared to unstimulated controls at gene level, with similar insignificant increases seen at secreted protein level (Figure 2A,B).

Ang‐2 expression was not regulated by cytokines from ASCs or MSCs under 21% oxygen at gene or protein level (Figure 2C,D), yet levels of Ang‐2 were significantly higher in ASCs under 5% O_2_ compared to 21% O_2_, and cytokines IL‐1β and TNFα significantly reduced expression of Ang‐2 at gene and protein level in ASCs under 5% O_2_ compared to unstimulated controls (Figure 2C,D).

FGF‐2 levels were not transcriptionally regulated by cytokines in ASCs or MSCs under 21% O_2_ (Figure 2E); however, secreted levels of FGF‐2 were significantly increased by TNFα in ASCs under 21% O_2_ compared to unstimulated controls (Figure 2F). Significant upregulation of FGF‐2 at gene level was evident in ASCs following cytokine stimulation (Figure 2E); however, secreted levels of FGF‐2 remained unaffected by low oxygen and cytokines (Figure 2F).

VEGF‐A was not regulated by cytokines under 21% O_2_ at gene (Figure 2G) or protein level (Figure 2H). Under 5% O_2_, VEGF‐A levels were significantly upregulated by IL‐1β and TNFα in ASCs at gene and protein level (Figure 2G‐H). Additionally, MSCs significantly upregulated gene and protein levels of VEGF‐A in response to IL‐1β but not TNFα (Figure 2G‐H).

### 4.3 Neurotrophic factor expression in ASCs and MSCs in response to low oxygen and inflammatory cytokines

**Figure 3:**
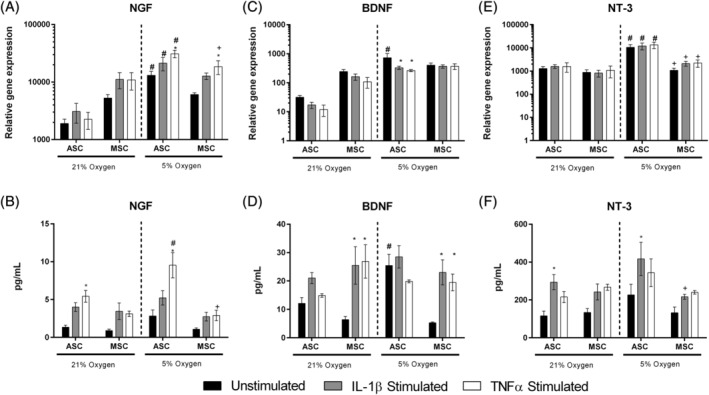
Gene and protein regulation of neurotrophic factors in human donor‐matched ASCs and MSCs exposed to cytokines IL‐1β or TNFα under 21% or 5% oxygen. Neurotrophic factors NGF (A,B), BDNF (C, D) and NT‐3 (E, F) mRNA expression (A,C,E) and secreted protein levels (B,D,F) in human ASCs and MSCs stimulated with either IL‐1β or TNFα under different oxygen concentrations for 48 hours. Gene expression was normalized to that of reference gene 18S. Values represent the mean ± SEM. Statistical significance determined using two‐way ANOVA with Tukey's multiple comparisons test, significance reported if *P* ≤ .05 (* = significant compared to unstimulated control within the same group; # = significant compared to corresponding sample in 21% oxygen; + = significance between MSCs and ASCs of the same treatment; n = 3)

Here, we aimed to assess the effect of inflammatory factors and different oxygen levels on the expression and secretion of several neurotrophic factors from donor‐matched MSCs. While NGF gene expression was not regulated by cytokines under 21% O_2_ in ASCs or MSCs, ASCs significantly increased its expression when cultured in 5% O_2_ (Figure 3A)_._ Under hypoxia both ASCs and MSCs responded to TNFα stimulation with an increase in NGF, but a smaller increase was observed following IL‐1β stimulation, yet this change failed to reach significance (Figure 3A). At protein level, NGF secretion into the culture media was significantly increased following TNFα stimulation in both 21% and 5% O_2_ from ASCs; however, levels were significantly higher from ASCs under 5% O_2_ compared to 21% O_2_ (Figure 3B).

BDNF gene expression levels were significantly higher in ASCs under 5% O_2_ compared to ASCs in 21% O_2_ (Figure 3C). Gene expression was significantly downregulated by IL‐1β and TNFα stimulation in 5% O_2_ compared to the corresponding unstimulated control (Figure 3C). BDNF expression from MSCs was not altered by cytokine stimulation or O_2_ levels at gene level (Figure 3C), yet secreted levels of BDNF were significantly upregulated by IL‐1β and TNFα in MSCs at both 21% and 5% O_2_ compared to unstimulated controls (Figure 3D). BDNF secretion from ASCs was not altered by cytokine stimulation, yet unstimulated ASCs under 5% O_2_ secreted significantly higher levels of BDNF than unstimulated ASCs under 21% O_2_ (Figure 3D).

Gene expression of NT‐3 was not altered by cytokine stimulation in either cell type (Figure 3E), yet levels of secreted NT‐3 were significantly increased upon IL‐1β stimulation in ASCs at 21% and 5% O_2_ (Figure 3F).

### 4.4 Neuropeptides were transcriptionally regulated by low oxygen in ASCs and MSCs

**Figure 4:**
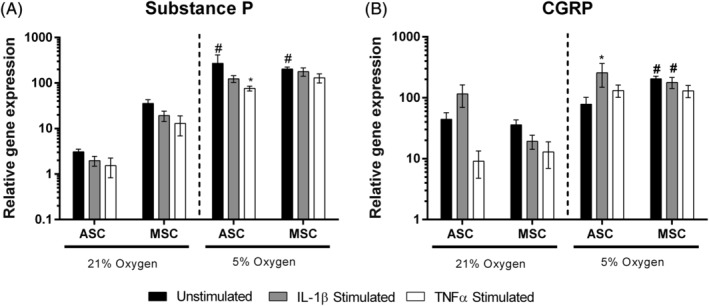
Regulation of neuropeptides in human donor‐matched ASCs and MSCs exposed to cytokines IL‐1β or TNFα under 21% or 5% oxygen. Substance P (A) and CGRP (B) mRNA expression in human ASCs and MSCs stimulated with either IL‐1β or TNFα under different oxygen concentrations for 48 hours. Gene expression was normalized to that of reference gene 18S. Values represent the mean ± SEM. Statistical significance determined using two‐way ANOVA with Tukey's multiple comparisons test, * = *P* ≤ .05 (* = significant compared to unstimulated control within the same group; # = significant compared to corresponding sample in 21% oxygen; + = significance between MSCs and ASCs of the same treatment; n = 3)

Here, we aimed to investigate the potential of MSCs to produce pain related peptides in response to low oxygen and inflammatory cytokines, which could subsequently lead to sensitization of ectopic sensory nerve fibers, or increase inflammatory cytokine production from NP cells as previously shown. Substance P gene expression was significantly higher in unstimulated ASCs and MSCs cultured under 5% O_2_ compared to 21% O_2_ (Figure 4A). Expression of substance P from ASCs under 5% O_2_ was downregulated by TNFα stimulation (Figure 4A). Substance P expression was not altered in MSCs in response to cytokine stimulation (Figure 4A). Substance P secretion from both ASCs and MSCs were below the limit of detection (< 9.76 pg/mL) regardless of oxygen levels or cytokine stimulation (data not shown).

CGRP expression remained unaffected by cytokines in either ASCs or MSCs under 21% O_2_ (Figure 4B). Gene expression levels of CGRP were significantly upregulated by IL‐1β in ASCs under 5% O_2_ compared to unstimulated controls (Figure 4B). CGRP levels were significantly higher in MSCs under 5% O_2_ compared to MSCs under 21% O_2_ (Figure 4B).

## 5 DISCUSSION

Cell‐based therapies aim to intercept disease progression through cellular repopulation and exploitation of the immunomodulatory capacity of ASCs and MSCs,^25^ yet it remains unclear which source of stem cell provides superior functionality for regeneration of the IVD. Interestingly, all cellular and biological therapeutics to date fail to address the presence of ectopic sensory nerve fibers within a subset of painful degenerate IVDs. Of the published clinical trials thus far investigating cell therapies for regeneration of the IVD, several report a cohort of non‐responders; yet it remains unclear as to why these patients do not show any improvements in pain and disability scores. With several reports highlighting the regenerative capacity of ASCs and MSCs in neural and vascular pathologies,^26^ a likely cause of persistent pain in patients with innervated IVDs is the stem cell‐mediated survival and migration of neural and vascular networks following delivery.

To the best of our knowledge, this is the first report addressing the neurovascular response of human donor‐matched ASCs and MSCs to two characteristic features of degenerate discs; low oxygen and pro‐inflammatory cytokines. It is widely acknowledged that vascularization is accompanied by neural ingrowth within the IVD and so it is important to demonstrate how stem cell‐therapies may promote the persistence of these structures following implantation.^27^, ^28^, ^29^ Therefore, it is important to demonstrate how stem cell‐therapies may promote the persistence of these structures following implantation. Here, we report constitutive expression of several potent angiogenic and neurotrophic factors from ASCs and MSCs and their regulation by low oxygen and cytokines IL‐1β and TNFα.

Vascularization of the degenerate IVD is thought to occur due to alterations in the matrix composition and resultant changes in intradiscal pressure, permitting the survival and inward migration of NGF expressing microvascular blood vessels, and concomitant sensory nerve fibers. Comparison studies investigating the therapeutic potential of ASCs and MSCs following hindlimb ischemia highlight inconsistencies in their ability to re‐vascularize damaged tissue. Studies by Bortolotti et al report effective perfusion and functional recovery following ischemia by MSCs, reporting increased levels of factors involved in vessel remodeling and stabilization, although no differences in Ang‐1 or VEGF‐A were reported.^30^ In contrast, reports by Kim et al demonstrated superior recovery of blood flow in an ASC treated group, with conditioned medium from ASCs also demonstrating heightened tube formation in vitro compared to MSCs.^31^ Other studies largely conclude that the therapeutic potential of stem cells is attributed to their differentiation into vascular endothelial cells but also to their ability to produce various angiogenic factors, including VEGF and FGF‐2.^31^, ^32^, ^33^ In the current study, we report increased transcriptional and translational expression of pro‐angiogenic VEGF‐A in response to reduced oxygen in ASCs and MSCs with higher levels evident in ASCs. We also report increased secretion of VEGF‐A from ASCs stimulated with IL‐1β under 5% oxygen. This study demonstrated significantly higher levels of other pro‐angiogenic factors including Ang‐1, Ang‐2, and FGF‐2 at gene level from ASCs when exposed to 5% oxygen compared to 21% oxygen. The presence of Ang‐1 was documented in 2012 by Sakai et al who determined Ang‐1 mRNA expression from disc progenitor cells as well as secreted Ang‐1 within the disc cell culture medium.^34^ The proposed role of angiogenic factors within a region that is largely avascular surrounds the maintenance and survival of native NP cells that have constitutive expression of HIF1α. Similarly, reports of FGF‐2 localization within the cells of IVD suggest roles for these factors in regulation and survival of NP cells under such hostile conditions.^35^ The increased levels of angiogenic factors from ASCs and MSCs in response to an environment mimicking that of the target tissue could result in neo‐angiogenesis and stimulation of preexisting blood vessels within the damaged tissue; in turn stimulating concomitant nerve fiber ingrowth.

Neurotrophic factors are associated with the survival, proliferation and migration of neurons, and increased levels of NGF, BDNF, and NT‐3 were evident in the present study from both ASCs and MSCs. NGF and NT‐3 were transcriptionally and translationally upregulated in hypoxic cultured ASCs compared with MSCs, with significantly increased secretion into the culture medium following TNFα conditioning. Previous studies by Redondo‐Castro et al reported native levels of trophic factors from MSCs under 21% oxygen.^36^ The authors assessed the response of MSCs to IL‐1β and TNFα stimulation in vitro; and in agreement with this study failed to demonstrate cytokine‐mediated NGF secretion. Interestingly however, we report significant TNFα‐mediated upregulation of NGF and IL‐1β‐mediated increases in NT‐3 from ASCs. MSCs but not ASCs increased secretion of BDNF following IL‐1β and TNFα stimulation. It has been hypothesized that ASC differentiation into a neuronal phenotype is enhanced when expression levels of BDNF and NT‐3 are increased simultaneously.^37^ This was demonstrated by transfection studies, showing increased ASC‐neuronal differentiation in BDNF and NT‐3 overexpressing ASCs alone and in combination. The authors hypothesized this may be due to concurrent increases in their tyrosine kinase receptors.^37^ Together with the data presented in this study, it could be further hypothesized that ASCs may alter their receptor profiles under different oxygen tensions and therefore become more responsive to cytokines, eliciting increased neurotrophic capabilities compared to MSCs.

Neuropeptide substance P is recognized as a pleiotropic molecule able act on sensory nerve fibers as a classical nociceptor, but more recently reported for its ability to enhance the stem cell phenotype and homing of MSCs to the site of injury.^38^ Neuropeptides are produced by degenerate NP cells^39^, ^40^, ^41^, ^42^, ^43^ and their regulation by pro‐inflammatory cytokines IL‐1β and TNFα is well documented.^40^, ^41^ Here, we report significant transcriptional regulation of substance P by low oxygen in ASCs yet this was not evident in the conditioned medium. Induction of substance P gene expression and release has been documented previously in transdifferentiated neural MSCs stimulated with IL‐1α,^44^ suggesting the induction of a neural phenotype is required for the production and release of substance P into the environment.

**Figure 5:**
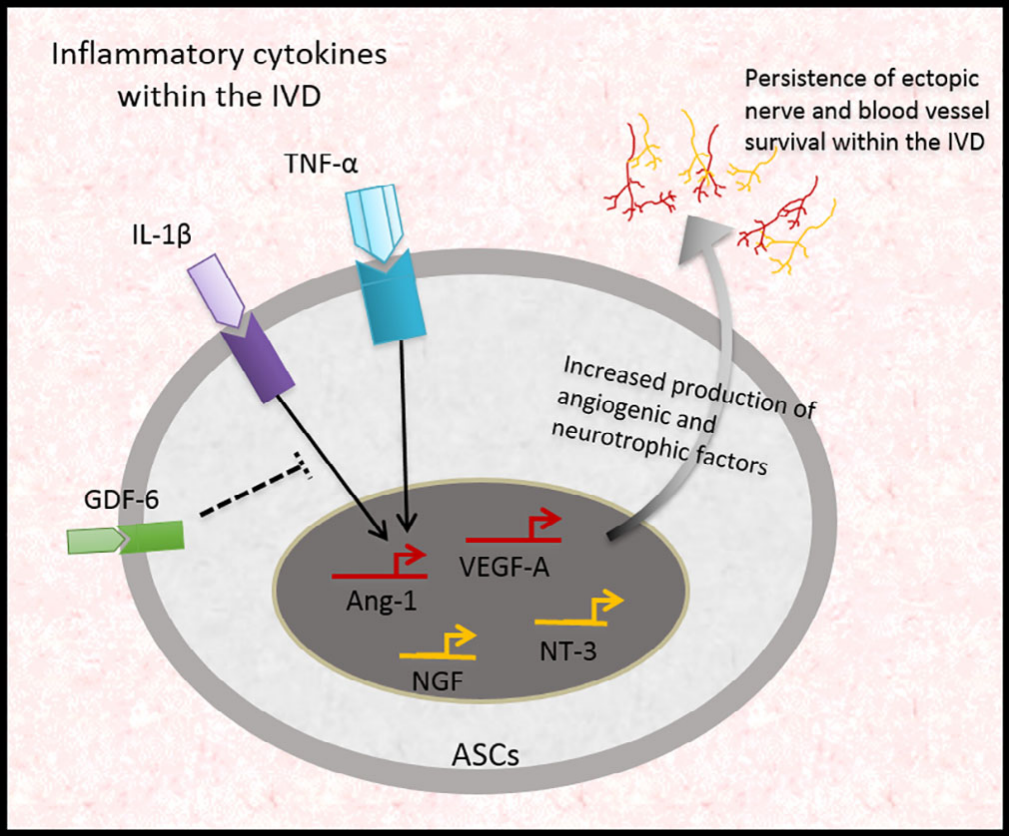
The role of inflammatory cytokines on ASCs under low oxygen environment and their implications in regulating neurovascular persistence and survival within the degenerate IVD. The highly inflammatory nature of the degenerate IVD will expose delivered stem cells to multiple pro‐inflammatory cytokines. TNFα and IL‐1β increased expression of neurotrophic and angiogenic factor expression and secretion from ASCs under low oxygen compared to MSCs. The production of these neurovascular factors may lead to the sensitization of ectopic sensory nerve fibers within the degenerate IVD resulting in sustained pain in these patients. Therefore, we hypothesize that pre‐stimulation or pretreatment of ASCs with GDF‐6 may induce an NP‐like phenotype and reduce their responsiveness to the degenerate microenvironment upon delivery

The data presented here highlights increased responsiveness of ASCs to cytokine stimulation under low oxygen by enhancing secretion of factors involved in neurogenesis and neo‐angiogenesis within the IVD; a process that could lead to persistent discogenic pain. Although we report that naïve ASCs have a greater potential to increase their expression and secretion of factors involved in nerve survival and vascularization under hypoxic and inflammatory conditions, the minimally invasive availability of these stem cells is still an attractive source for therapy. ASCs retrieved from adipose tissue following liposuction plastic surgeries have been shown to be up to 500 times more prevalent than MSCs when comparing an equivalent volume of tissue.^45^ ASCs may benefit from being predifferentiated or preconditioned prior to delivery to reduce their responsiveness to low oxygen levels and the inflammatory milieu (Figure 5). Clarke et al were the first to report the use of growth differentiation factor‐6 (GDF‐6) as a differentiation factor promoting NP‐like differentiation of ASCs in replicating matrix synthesis and gene expression of classical NP markers.^23^ It could therefore be hypothesized that GDF‐6 pre‐differentiated ASCs could enhance NP cell repair as well as attenuating the inflammatory and neurovascular environment as recently reported by Miyazacki et al.^46^

While this study utilized monolayer culture of human MSCs, it forms a basis for future studies into the mechanistic actions of MSCs when exposed to inflammatory and oxygen depleted environments such as the IVD; and future studies should include 3D cultures of MSCs and NP cells to assess the interaction between different cell types following delivery and their effect on preexisting neural and vascular structures. The IVD is an extremely complex tissue undergoing progressive changes and so it is important to consider all aspects of the target tissue to ensure correct cell types and delivery systems are in place to ensure optimal success rates, including but not limited to a soft, hydrated 3D environment subjected to mechanical load. Therefore, further research should be invested into elucidating the mechanisms by which MSC‐based therapies aid regeneration of the IVD tissue, and avoid the promotion of ectopic structures that may hinder clinical outcomes.

## 6 CONCLUSION

To conclude, this is the first report to highlight fundamental differences in neurovascular profiles of donor‐matched ASCs and MSCs exposed to inflammatory conditioning under low oxygen tensions in order to provide insights into the importance of stem cell selection for regenerative therapies. This includes other tissues, which have a hypoxic and inflammatory environment in response to injury or disease and which conversely to IVD require angiogenesis and innervation for healing, for example, bone lesions. When considering therapies for regeneration of the IVD, selecting the optimal cell source to repair the tissue as well as preventing neo‐angiogenesis and ectopic sensory nerve fiber ingrowth is crucial to reducing discogenic pain and improving patient outcomes. These observations have allowed us to propose a neurovascular response network associated with cell delivery to the degenerating disc. It appears that NGF, NT‐3, VEGF‐A, and Ang‐1 are key players in this network, and future work should investigate the effect of the ASC/MSC secretome on native NP cells to ensure clinical efficacy of cell‐based regenerative therapies.

## CONFLICT OF INTERESTS

F.B. is a shareholder and director of Orbsen Therapeutics Ltd. F.B. is a shareholder of Osiris Therapeutics Inc. A.L.A.B., S.M.R., and J.A.H. declare that they have no competing interests.

## AUTHOR CONTRIBUTIONS

A.L.A.B. participated in the study design, data acquisition, analysis and interpretation of data, statistical analysis, and manuscript preparation. S.M.R. interpreted the data, reviewed, and edited draft versions including final manuscript draft. J.A.H. and F.P.B. participated in the study design, interpreted the data, reviewed, and edited draft versions including final manuscript draft. All authors have read and approved the final submitted article.
